# How much bilateral and multilateral climate adaptation finance is targeting the health sector? A scoping review of official development assistance data between 2009–2019

**DOI:** 10.1371/journal.pgph.0001493

**Published:** 2023-06-14

**Authors:** Tilly Alcayna, Devin O’Donnell, Sarina Chandaria

**Affiliations:** 1 Red Cross Red Crescent Climate Centre, The Hague, The Netherlands; 2 Centre on Climate Change & Planetary Health, London School of Hygiene & Tropical Medicine, London, United Kingdom; 3 Centre for Mathematical Modelling of Infectious Diseases, London School of Hygiene & Tropical Medicine, London, United Kingdom; 4 Health in Humanitarian Crises Centre, London School of Hygiene & Tropical Medicine, London, United Kingdom; Monash University, AUSTRALIA

## Abstract

Climate change is adversely affecting human health. Rapid and wide-scale adaptation is urgently needed given the negative impact climate change has across the socio-environmental determinants of health. The mobilisation of climate finance is critical to accelerate adaptation towards a climate resilient health sector. However, a comprehensive understanding of how much bilateral and multilateral climate adaptation financing has been channelled to the health sector is currently missing. Here, we provide a baseline estimate of a decade’s worth of international climate adaptation finance for the health sector. We systematically searched international financial reporting databases to analyse 1) the volumes, and geographic targeting, of adaptation finance for the health sector globally between 2009–2019 and 2) the focus of health adaptation projects based on a content analysis of publicly available project documentation. We found that health was largely a co-benefit, not the principal objective, within the projects. We estimate that USD 1,431 million (4.9%) of total multilateral and bilateral adaptation has been committed to health activities across the decade. However, this is likely an overestimate of the true figure. Most health adaptation projects were in Sub-Saharan Africa, with average project funding comparable to East Asia and the Pacific and the MENA region. Fragile and conflict affected countries received 25.7% of total health adaptation financing. The paucity of health indicators as part of project monitoring and evaluation criteria and the lack of emphasis on local adaptation were particularly significant. This study contributes to the wider evidence base on global health adaptation and climate financing by quantifying adaptation funds directed towards the health sector and revealing specific gaps in financing health adaptation. We anticipate these results will support researchers in developing actionable research on health and climate finance and decision-makers in mobilizing funds to low-resource settings with high health sector adaptation needs.

## Introduction

The health sector faces a dual challenge vis-à-vis climate change. Rapid and wide scale adaptation is needed to prepare for, minimise and potentially avoid impacts which are already adversely affecting human health and health systems [1–5]. Simultaneously, the health sector must rapidly ‘green’ to reduce its carbon footprint, as its activities contribute 4–4.9% of annual greenhouse gas emissions globally [3,6,7]. Adaptation to climate change within the health sector aims to reduce death and illness by improving the resilience of health infrastructure to shocks and stresses, utilising early warning systems for extreme weather events or climate-sensitive infectious diseases [4], enhancing disease surveillance, developing heat health action plans [8,9], and expanded mental health and psychosocial health (MHPH) care [10,11], amongst other activities [12,13]. Overall, health adaptation requires a system-wide integrated and transformational approach to reduce climate risk and cultivate high adaptative capacity within health systems. Moreover, health should be integrated as a cross-cutting principle across all sectors including food systems, livelihoods, social support structures, water and sanitation, amongst others [1].

As part of United Nations Framework Convention on Climate Change (UNFCCC) commitments, countries develop and regularly update Nationally Determined Contributions (NDCs) outlining the pathway to meet carbon reduction targets (e.g., as part of mitigation efforts) and identify priority areas for adaptation. 73% (135/185 countries) of NDCs include a reference of health, with 60 countries noting climate-related health outcomes or adaptation measures and 40 countries having detailed health adaptation plans [14]. A 2021 WHO analysis of 19 National Adaptation Plans (NAPs) found that all reviewed NAPs highlighted health as a “high-priority sector vulnerable to climate change” [15] with varying consideration taken to address highlighted health risks. Countries are increasingly prioritising climate change and health through the development of specific strategies and plans such as Health National Adaptation Plans (HNAPs) [16]. However, there remains a disconnect between written priorities and the direction of financial flows [2,17]. The 2021 WHO Health and Climate Change Global Survey found that whilst 51% of countries reported having a national health and climate change strategy or plan in place, less than a quarter of respondents had achieved high or very high levels of implementation [18]. Insufficient financing or budget was identified as a key barrier to reaching full implementation by 70% of responding countries [18].

The UNFCCC estimates that global health adaptation will require USD 26.8 to USD 29.4 billion in funding annually by 2050 [19]. Many low income countries are unable to provide basic health services, much less the investments needed to adapt services to unfolding climate risks [20]. The World Bank estimates that by 2050 Sub-Saharan Africa will incur 80% of the global health costs from increased case burden of malaria and diarrheal disease [21]. If health system adaptation is not prioritised, an increased health burden will contribute significantly to the both the economic cost and non-economic loss and damage attributable to climate change [22].

International climate finance will play an important role—alongside domestic government spending and public-private partnerships—in funding the unmet need for health sector adaptation. Across all sectors, mitigation receives significantly more public climate financing than adaptation [23]. Global tracking shows adaptation finance as having reached just USD 46 billion in 2019/2020 [24], significantly lower than estimates in the upper range of USD 140–300 billion needed for adaptation efforts by 2030 [25]. The 2018 Adaptation Gap Report, which took a special focus on health, concludes that “there is a significant global adaptation gap in health, as efforts are well below the level required to minimise negative health outcomes” [26]. To date, funding for adaptation projects that specifically address health are a minute percentage (<0.5%) of an already limited pool of adaptation funding [3,27]. The mobilisation of climate adaptation finance is an important mechanism by which to address climate change impacts on health in the most vulnerable countries. Therefore, it is essential to track funding flows and establish baseline measurements in order to better coordinate the use of scarce public resources.

The Lancet Countdown tracks health financing via an indicator (‘2.2.4 Health related-adaptation funding’ in 2021) [28–32]. These estimates are typically cited in reports from the WHO [18] or United Nations, for example, in the United Nations Environment Program Adaptation Gap Report 2018 [26]. The indicator is composed of two elements: first, spending on adaptation for health and health-related activities; and second, health adaptation funding from global climate financing mechanisms. Estimates are derived from the kMatrix Adaptation and Resilience to Climate Change dataset (a private dataset, which track financial transactions from insurance companies, financial sector, governments relevant to climate change adaptation) and the Climate Funds Update database (a public dataset, which tracks data on multilateral climate change funds) [29,33,34]. Successive Lancet Countdown reports estimate that health adaptation funding as a percentage of total adaptation financing has been increasing from 4.6% across 2015–2016 [28], 3.8% in 2017 [35], 5% across 2017–2018 [36], 5.3% across 2018–2019 [30], 5.6% in 2019–2020 [32], and 5.6% in 2020–2021 [31]. The Lancet Countdown also estimates health-related adaptation financing (i.e. projects that could support health and health-care adaptation in other sectors, such as agriculture, water, transport etc) which tends to be higher than health specific financing (13.3%-28.5% between 2017–2022) [28,30,35,36]. For international climate financing mechanisms, the Lancet Countdown draws on only multilateral donor information (from Climate Funds Update) [28,34] or specifically only from the Green Climate Fund [31].

Here, we seek to build on these previous estimates by extending the analysis of health adaptation funding from international climate financing mechanisms to track volumes of both multilateral donor adaptation funding and bilateral donor adaptation funding from publicly available datasets. We investigate two key questions. Firstly, what are the volumes of adaptation finance targeting the health sector globally in the previous decade (2009–2019), and which countries access this money? Secondly, what is the focus of the health adaptation projects funded in the past decade (2009–2019)? We build on previous examinations of international climate adaptation finance for the health sector in three key ways. First, by analysing a longer time series of adaptation finance data (2009–2019, a full decade). Second, by including a quantitative review of *bilateral* climate finance data in addition to multilateral climate finance data. Third, by reviewing both volumes of financing *and* the content of health adaptation projects through a qualitative content analysis of publicly available project documentation. Estimating volumes of funding since the 15^th^ Conference of the Parties (COP15) of the UNFCCC in Copenhagen in 2009 marks an important decadal baseline, as wealthier countries committed to collectively mobilising USD 100 billion per year with equal consideration for mitigation and adaptation by 2020 during this meeting [37]. This commitment was not met in 2020 and postponed to 2023 [38,39].

This study contributes to the wider evidence base on health adaptation funding from international climate financing gaps by providing geographical information on the volume of health adaptation funding globally and by examining the gaps in health adaptation activities. It is widely known that funding efforts are currently well below the level required to minimise negative health outcomes [26], and yet action is slow to mobilise. Our aim is to provide further evidence to support researchers in developing actionable research on health and climate finance and decision-makers in mobilizing funds to low-resource settings with high health sector adaptation needs.

## Materials and methods

### Search strategy

PRISMA-ScR guidelines [40] were adapted for this review of all adaptation projects in publicly available datasets. The two main online databases for project-level information on climate adaptation finance were searched. These were: Climate Funds Updates (CFU) which contains information on climate adaptation and mitigation projects funded by multilateral organisations [34] and the OECD-DAC ‘Rio Markers’ climate-related development finance database which contains information on climate adaptation and mitigation projects funded by bilateral donors and some multilateral organisations [41]. The search was completed from September 2021 to July 2022. 2009 to 2019 OECD-DAC data was downloaded. 2003 (the year the database was approved) to 2021 data from CFU was downloaded. These downloaded results were stored in Microsoft Excel files. To ensure consistency between the two databases only projects between 2009–2019 were analysed, to provide a time series of a decade. Projects were extracted to a separate Excel sheet for further analysis if they were tagged as ‘health’ according to the database categorisation or via a keyword search of project titles for ‘health specific’ projects (see inclusion criteria) carried out by the research team (see S1 Data). Duplicates and irrelevant projects were removed. Funding amounts for duplicates between CFU and OECD databases were averaged. Proposals or evaluations (i.e., project documentation) of the relevant projects were then manually searched for via an internet search of donor databases. Three reviewers (TA, DO, SC) screened the full texts independently and disagreements were resolved by consensus.

### Inclusion criteria

The following inclusion criteria were applied: projects must have clear climate objectives; projects must have been awarded funding between 2009 and 2019; projects must be a health-related adaptation project; and funding must have been awarded by multilateral or bilateral donors (i.e., not private). Projects were assumed to be adaptation if they were labelled as such by the databases (see S1 Text). For example, in the OECD-DAC ‘Rio Markers’ are used for climate change mitigation or adaptation projects [42]. ‘Only ‘principal’ labelled adaptation projects in the OECD-DAC were retrieved as these are official development assistance (ODA) projects that have been explicitly designed with climate adaptation objectives. All projects on the CFU database have strict climate objectives and as such were treated as ‘principal’ equivalent. Projects were included if they were *tagged* by the OECD-DAC and CFU database as ‘health’. For the OECD-DAC database the following *health tag purpose codes* were used: 1.2 Health, 1.2.a General Health, and 1.2.b. Basic Health (12110, 12182, 12220, 12230, 12250, 12261, 12262, 12263, 12281). For CFU, the ‘health tag’ is categorized using the DAC120- tag which includes all DAC health markers (1.2 Health, 1.2.a General Health, 1.2.b Basic Health). Additionally, projects were included if they contained one of the following health specific keywords in their title or abstract (Table 1). Projects were excluded for the qualitative analysis if they did not have a direct health-specific objective (i.e., the health objective was not explicitly indicated in the activity documentation), if project documentation could not be retrieved, or if the documentation was not in English (see Limitations).

**Table 1:** Search terms using health tags and health specific keyword search.

	OECD-DAC database	CFU database
**Health tag**	Health purpose code: 1.2 Health, 1.2.a General Health, and 1.2.b. Basic Health (12110, 12182, 12220, 12230, 12250, 12261, 12262, 12263, 12281	Health tag: DAC120- tag which includes all DAC health markers (1.2 Health, 1.2.a General Health, 1.2.b Basic Health)
Keywords	Health OR Epidemic OR Surveillance OR Disease* OR ‘Non-communicable disease’ OR Diabetes OR ‘Heart disease’ OR Obesity OR Mortality OR Hospital OR ‘Primary care’ OR Nursing OR Transmission OR Nutrition OR Malnutrition OR Psychosocial OR Waterborne OR water-borne OR Diarrhea* OR Cholera*OR Hepatitis OR Leptospirosis OR Vectorborne OR vector-borne OR ‘vector borne’ OR Malaria* OR Dengue OR Zika OR Chikungunya OR Zoonot* OR ‘West Nile Fever’ OR ‘Rift Valley Fever’ OR Ebola OR Tickborne OR tick-borne OR ‘tick borne’ OR Heat*	

### Data cleaning

In order to avoid double-counting the same project (due it being listed on both CFU and OECD databases) and over-counting the number of projects in a given country (due to the same project listed across multiple years due to funding being released in tranches), these repeat entries were identified and consolidated. Firstly, the funding amounts for duplicates (identifying by having the same title, year, donor, recipient country, and implementing agency) between CFU and OECD were averaged. Secondly, in cases where a project (with the same title, donor, recipient country, implementing agency) had multiple entries over consecutive years with different funding amounts, the original project documents were located, and tranche funding was assumed if the total funding allocated in the project documents aligned with the sum of the entries listed in the dataset. These entries were consolidated into one entry with a sum of the total funding. For entries that had the same title, donor, recipient country, implementing agencies but different funding amounts over multiple years, but where documents were not located, they were assumed to be representing the same project with tranche funding if the funding was received in consecutive years or with no more than two years between funding receipt. These entries were consolidated into one entry with a sum of the total funding. Thirdly, for entries that had the same title, donor, recipient country, and implementing agency but had funding more than five years apart (e.g., 2014 and 2019), project documents were located and used to clarify if this was the same project (and therefore consolidated) or separate phases of a project with different proposals (and therefore left as separate entries). Where project documentation was not located, entries were left separate. Finally, for entries that had minor variations in title (e.g., Rural Water for Sudan-Darfur and Rural Water for Sudan-East) but funding was provided in the same year (e.g., 2017), original project documentation was located and checked. If these entries were clearly part of the same programme, then they were consolidated with a sum of total funding. If project documentation was not located and/or funding was received in different years alongside minor variations in title, then entries were left separate.

### Quantitative data analysis

A quantitative descriptive analysis of the retrieved projects was carried out to describe the total number of health-adaptation projects within overall adaptation projects; funding trends over the past decade; geographical distribution of health-adaptation projects; average project funding per region; and who the major donors were (bilateral/multilateral) and funding amount. Summary statistics and pivot tables were generated using Excel.

### Content analysis

A content analysis matrix was developed for the systematic extraction of data from retrieved multilateral project documentation (proposals and evaluations). Data were extracted for the following variables: Project title; Donor; Implementing partner; Country; Region; Country classification (high income, middle income, low income etc); Funding amount (committed, 2019 equivalent); Funding type (grant, loan); Duration; Document type (proposal, evaluation etc); Health tag or keyword; Target population; Climate shock or stressor addressed; Health focus (e.g. non communicable disease, infectious disease, health system, etc); Intended health outcome; Main program activity; Health indicator.

Following this data extraction, projects were then classified as health principal, health significant, or not health focused. Projects were tagged as *health principal* if the main project aim, objectives, and metrics were directly tied to the health sector (i.e., health system capacity building) or the prevalence of health conditions (i.e., reducing vector-borne disease). Projects were tagged as *health significant* if the main project aim, objectives, and metrics were not directly tied to the health sector, but a strong correlation was made between the activities and a health outcome. The health outcome should be both defined and measured in order for the project to be tagged as *health significant*. For example, livelihood and agricultural programming that showed a clear, measured relationship between the activities and a reduction in food insecurity or malnutrition would be tagged as health significant. Projects were tagged as *not health focused* if a connection was not made between the main program activities and a health outcome. For example, a livelihood programme that did not make the connection between the programme activities and a measured potential health outcome, irrespective of whether it could have a potential health benefit, was tagged as not health focused.

## Results

**Fig 1:**
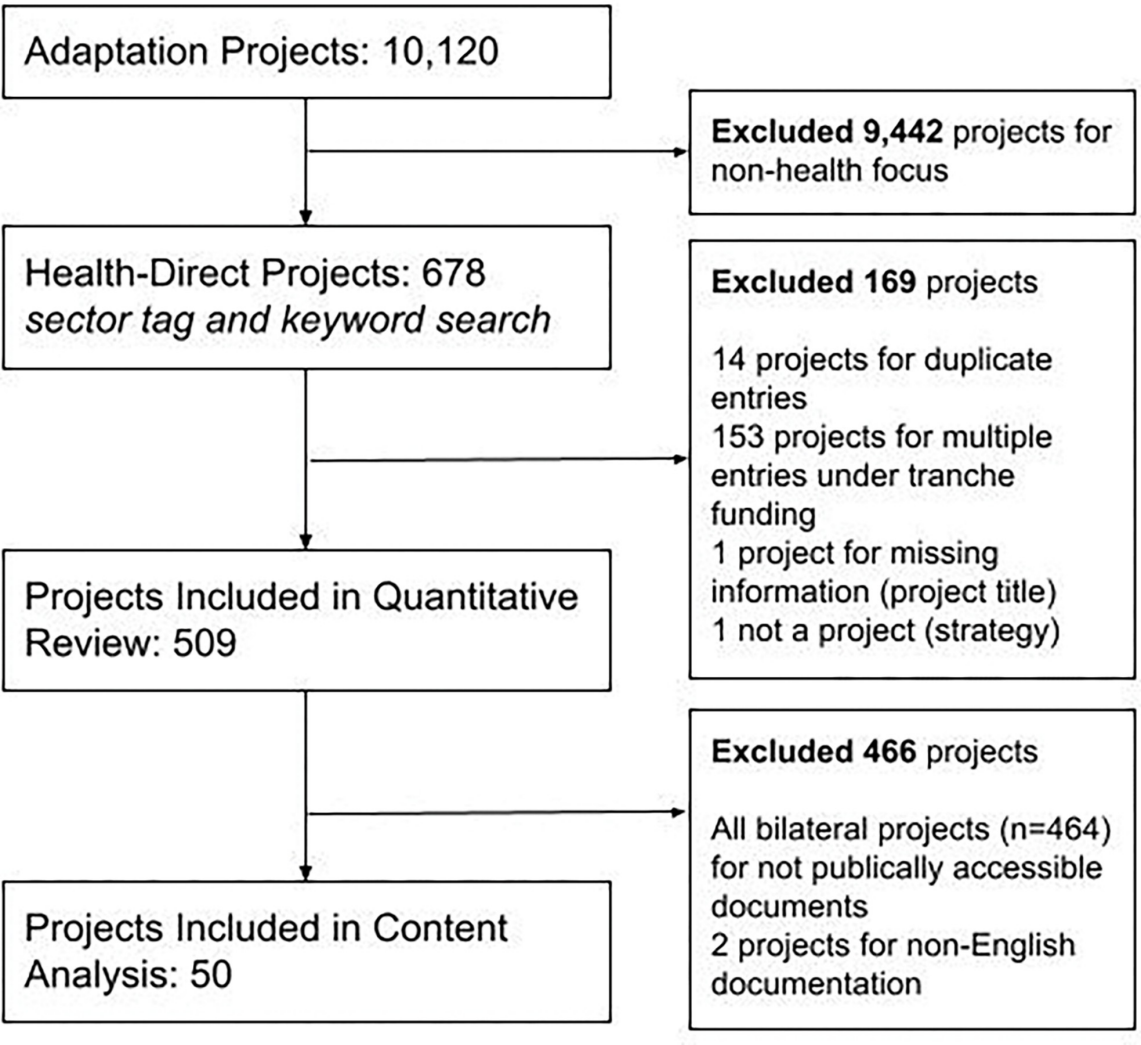
PRISMA-ScR reporting flow chart. Projects were excluded at three stages throughout the analysis.

From the 10,120 multilateral and bilateral adaptation projects retrieved from the CFU and OECD databases between 2009 and 2019, 678 entries were health adaptation projects. After applying the exclusion criteria, 509 health adaptation projects remained for quantitative analysis (See Fig 1).

### Volumes of funding

We estimate that USD 1,431 million (4.9%) of multilateral and bilateral international adaptation finance has been committed to health activities in a decade of climate adaptation financing (2009–2019) (see Table 2). Multilaterals funded 52 health projects worth USD 521.8 million (1.8% of total adaptation financing or 5.7% of total multilateral adaptation funding). Bilaterals funded 457 health projects, worth USD 910.1 million (3.1% of total adaptation financing or 4.6% of total bilateral adaptation funding).

**Table 2:** Volumes of global international adaptation finance and corresponding number of projects (2009–2019) derived from OECD and CFU databases.

Timeframe (2009–2019)		Number of projects n	Volume of funding USD million (%)
**Total Adaptation (General)**		**10,120**	**29,029.4 (100)**
	Multilateral Adaptation (General) Multilateral Adaptation (Health) Multilateral Adaptation (Health ‘Principal’) Bilateral Adaptation (General) Bilateral Adaptation (Health) **Total Adaptation (Health)**	1,725 52 11 8,395 457 **509**	9,092.7 (31.3) 521.8 (1.8) 103.9 (0.4) 19,936.7 (68.7) 910.1 (3.1) **1,431 (4.9)**
**Multilateral Adaptation (General)**		**1,725**	**9,092.7 (100)**
	Adaptation (Health) Adaptation (Health ‘Principal’)	52 10	521.8 (5.7) 46.21 (0.5)
**Bilateral Adaptation (General)**		**8,395**	**19,936.7 (100)**
	Adaptation (Health)	457	910.1 (4.6)

## Donor funding

**Fig 2:**
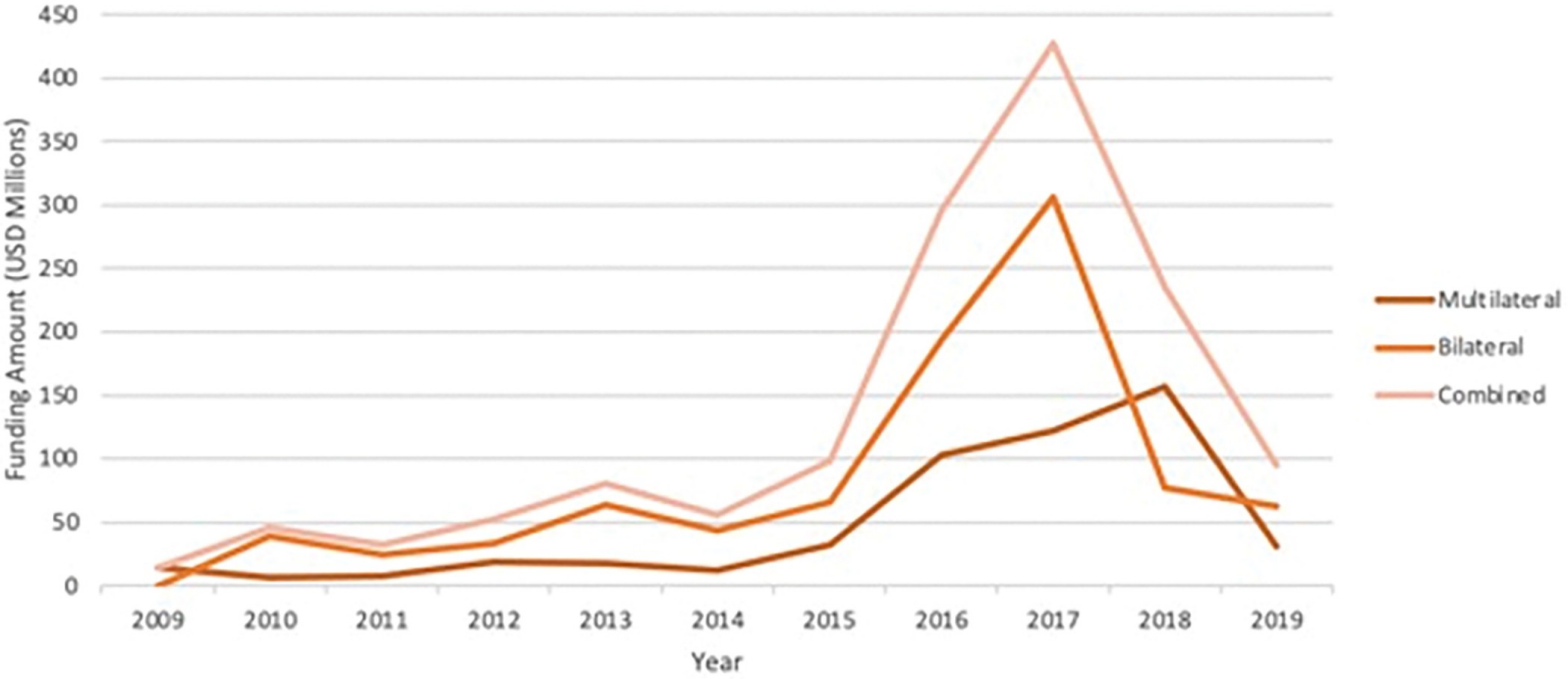
Volumes of health-related climate adaptation finance, 2009–2019. Source: OECD-DAC CRS database and CFU database.

Donors contributed a small amount (less than USD 100 million) of funding to health prior to 2015. In 2015, multilateral and bilateral contributions towards health adaptation increased substantially (reaching USD 400 million in 2017) (see Fig 2). However, this increase was not maintained and in 2018 returned to amounts on par with pre-2015 levels. This increase in funding in the years following 2015 could perhaps be linked to the adoption of the Paris Agreement at COP21 or the beginning of project funding in the same year by GCF, with the subsequent lull potentially attributable to changing political goals around climate financing by key government funders.

More than half (59.7%) of all health adaptation finance was provided by three donors (see Table 3). EU institutions contributed 26.06% (USD 373.1 million) of total funding, followed by the Green Climate Fund (GCF) (21.93%, USD 314.0 million) and the United States (11.68%, USD 167.3 million). The EU institutions and the GCF both provided the highest average amount of funding per project (USD 31.1 million and USD 26.2 million, respectively). The United States had a high number of projects compared to other donors (128 projects 2009–2019), but relatively low funding per project (USD 1.3 million).

**Table 3:** Top 20 donors ranked by volume of health adaptation finance and corresponding number of projects (2009–2019) derived from OECD and CFU databases.

Rank	Donor	Volume Funding USD millions (%)	Total projects n	Average per project USD millions
	**All**	**1431.9 (100)**	**509**	**2.8**
**1**	EU Institutions (excl. EIB)	373.1 (26.1)	12	31.1
**2**	Green Climate Fund (GCF)*	314.0 (21.9)	12	26.2
**3**	United States	167.3 (11.7)	128	1.3
**4**	Adaptation Fund (AF)*	96.7 (6.8)	15	6.4
**5**	Germany	81.6 (5.7)	35	2.3
**6**	Ireland	51.3 (3.6)	81	0.6
**7**	Norway	45.9 (3.2)	23	2.0
**8**	Sweden	44.9 (3.1)	11	4.1
**9**	Global Environment Facility (GEF)*	35.4 (2.5)	7	5.1
**10**	United Kingdom	29.9 (2.1)	21	1.4
**11**	Switzerland	28.3 (2.0)	10	2.8
**12**	Special Climate Change Fund (SCCF)*	21.9 (1.5)	5	4.4
**13**	Adaptation for Smallholder Agriculture Prog.*	20.5 (1.4)	3	6.8
**14**	France	19.8 (1.4)	12	1.7
**15**	Least Developed Countries Fund (LDCF)*	18.2 (1.3)	4	4.6
**16**	Korea	13.4 (0.9)	4	3.3
**17**	Spain	12.1 (0.8)	22	0.6
**18**	United Arab Emirates	10.8 (0.8)	1	10.8
**19**	Canada	8.9 (0.6)	46	0.2
**20**	Australia	7.0 (0.5)	17	0.4

*multilateral funds are supported by country contributions. Individual country contributions to funds such as the GCF and GEF are not represented in this table.

Overall, 99% of projects were funded by grants (100% of bilateral and 94% of multilateral). Three multilateral projects were funded partially by grants alongside loans or equity financing. Many countries also contributed financing via multilaterals such as the GCF or GEF.

### Implementing agencies

Donor country based NGOs accounted for 27% (n = 139) of all implementing agencies. This was followed by donor governments (13%, n = 65), UN agencies such as UNDP, UNEP, UN Population Fund, WHO, FAO, IOM, IUCN, UNICEF (12%, n = 61), and recipient governments (11%, n = 58). The remaining implementing agencies were comprised of non-donor country-based NGOs, academic/research institutions, or multilateral banks (such as: the African Development Bank; the Asian Development Bank) amongst others.

### Geographic distribution of funding

**Fig 3:**
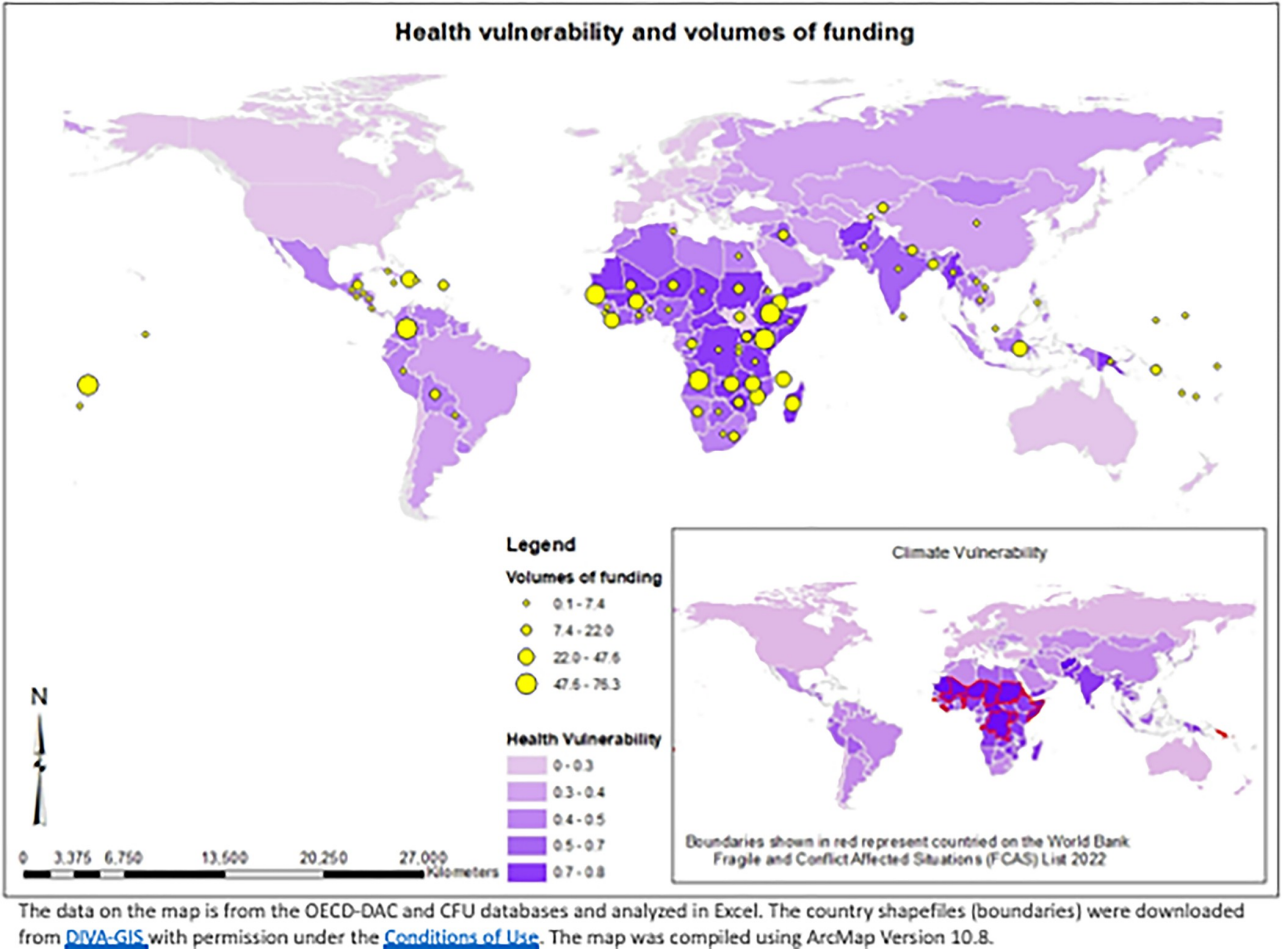
Comparison of the geographic distribution of climate and health vulnerability (according to ND-Gain Index) with health adaptation financing (2009–2019).

Climate adaptation financing for health has not consistently targeted countries with the highest level of health or climate vulnerability (according to the ND-GAIN Index) (see Fig 3). This index calculates health vulnerability as a composite indicator using “projected change in deaths from climate induced diseases, projected change in length of transmissions season of vector-borne diseases, dependency on external resources for health services, informal settlement population, medical staff, access to improved sanitation facilities” and climate vulnerability as the “propensity or predisposition of human societies to be negatively impacted by climate hazards” [43]. Certain countries in Sub-Saharan Africa (SSA) with high vulnerability to climate change and within the health sector (for example, the Democratic Republic of the Congo or Tanzania) have not received any health adaptation funding to date.

A regional analysis indicates that Sub-Saharan Africa (SSA) has received the largest proportion of funding and was the location of the highest number of health adaptation projects (61.8% of funding and 313 projects) from donors (multilateral and bilateral). However, average combined funding from multilaterals and bilaterals per project was comparable between SSA (USD 2.83 million), East Asia and Pacific region (USD 2.9 million), and Middle East and North Africa (MENA) (USD 2.8 million). Latin America and the Caribbean (LAC) received substantially higher per project funding (USD 4.1 million), whilst South Asia received substantially lower per project funding (USD 1.2 million) (see Table 4). Overall, 126 health adaptation projects were funded in Fragile and Conflict Affected Situations (FCS) amounting to USD 367.7 million (25.7% of total health adaptation financing). The majority of these projects (n = 112) were in SSA, followed by East Asia and the Pacific (n = 8), and LAC (n = 4) (see Table 4).

**Table 4:** Volumes of health adaptation financing (2009–2019) by region, average funding per project, and volumes of health adaptation channelled to fragile, and conflict affected situations (FCS) in the regions.

Region	Volumes financing	Average per project	FCAS Volumes financing
	USD million (%) n = projects	USD million	USD millions n = projects
**Grand Total**	**1431.9 (100)** **n = 509**	**2.8**	**367.9** n = 126
Sub-Saharan Africa (SSA)	885.3 (61.8) n = 313	2.8	291.2 n = 112
Latin America and the Caribbean (LAC)	223.0 (15.6) n = 54	4.1	47.6 n = 4
East Asia and Pacific	193.4 (13.5) n = 67	2.9	8.3 n = 8
Global or unspecified	55.5 (3.9) n = 29	1.9	0 n = 0
South Asia	37.4 (2.6) n = 32	1.2	0 n = 0
Middle East and North Africa (MENA)	27.8 (1.9) n = 10	2.8	20.7 n = 2
Central Asia	9.5 (0.7) n = 3	3.2	0 n = 0
Europe	0.01 (0.0) n = 1	0.01	0 n = 0

### Content analysis of multilateral project documentation

Of the 509 projects included in the quantitative analysis, 50 multilateral funded projects with publicly available project documentation met the inclusion criteria for content analysis. Forty percent of projects focussed on extreme weather events (such as floods, droughts, tropical cyclones, heatwaves, etc) (n = 20), whilst 54% included climate change generally (n = 27). Two projects were not specific about the climate change variables of focussed and one project focused on land salinisation. In general, most projects were focused on farmers (smallholders), agro-pastoralists, or fisherfolk (n = 16).

Health was the principal aspect of just 10 projects, focussing on infectious disease, health systems and disease surveillance. This corresponds to USD 45.9 million in funding (0.5% of multilateral adaptation finance or 0.2% of total adaptation finance). ‘Health principal’ projects sought to: strengthen the capacity of health systems; support the development of Health National Adaptation Plans or include health in National Adaptation Plans; develop health information systems and early warning systems that include health risks; improve policy and governance on climate change impacts on health; improve capacity of health workforce to deal with climate change impacts; and improve disease control and awareness. Most of these projects operated at the national level. Eight out of the ten ‘health principal’ projects were global or regional in nature. In terms of regional distribution seven were in Asia Pacific, one was unspecified, and two were in Sub-Saharan Africa. The main activities revolved around information, knowledge, and capacity building, therefore many of the proposals did not outline specific health indicators (e.g., a reduction of water-borne disease by a certain amount).

Health was a significant aspect in 25 projects (50%) wherein the proposal clearly defined a connection between the main activities and health outcomes. Health benefits of the project were both defined and measured. Ten of these ‘health significant’ projects focused on livelihood-based programming including food security or nutrition. The remaining projects included a WASH component (for water-borne diseases and water security) or generally improving wellbeing via health awareness raising. The majority of the health-relevant indicators included measures of food insecurity using established scales, number of meals, or number of people with access to WASH infrastructure or clean water.

30% of the projects (n = 15) were classified as ‘not health focused’ as a connection between health and the program aim was not made in the proposal, and health outcomes were not evaluated or measured. The program could still have a wider public health co-benefit. These projects commonly had main activities around animal health, disaster risk reduction, water infrastructure, or land management.

## Discussion

This scoping review re-emphasises a significant contradiction in the current climate adaptation landscape: that while donors agree that the climate crisis is a health crisis this is yet to be reflected in climate adaptation financing. Our findings contribute to previous efforts to track international climate finance for health by analysing publicly available datasets for bilateral adaptation finance in addition to multilateral adaptation finance, as the latter has solely been used for global estimates from previous studies [28]. First, we examined the volume and geographic targeting of climate adaptation finance. We estimate that globally USD 1,431 million (4.9%) of total multilateral and bilateral international adaptation finance has been committed to health activities in a decade of climate adaptation financing (2009–2019). Most health adaptation projects were in Sub-Saharan Africa, with average project funding comparable to the East Asia, Pacific and MENA region. Countries which are categorised as fragile and conflict affected situations received 25.7% of total health adaptation financing. Second, we assessed the focus of the health adaptation projects funded in the past decade (2009–2019). Within multilateral funding, the number of projects in which health was the principal focus corresponded to only about 0.5% of total multilateral adaptation financing or 0.2% of total adaptation financing. Thus, a clear gap in health adaptation financing for health persists.

Our examination of the volume of health adaptation financing offers a new estimate for bilateral funding and reveals some differences to previous estimates. We found that bilateral donors funded 457 projects, worth USD 910.1 million, and multilateral donors funded 52 health projects, worth USD 521.8 million. We were only able to compare our estimates for multilateral health adaptation funding, as previous analyses have largely focused on multilateral finance flows. We found multilateral projects tagged as health accounted for USD 521 million (5.7%) of total multilateral financing (USD 9.0 billion) whilst the Lancet Countdown 2021 found USD 711 million (13.9%) of total multilateral financing (USD 5.1 billion) went to health tagged projects [3]. This difference is likely due to methodological dissimilarities: a different time frame under analysis (2009–2019 vs 2018–2020, respectively), the use of different databases (CFU & OECD and CFU & KMatrix, respectively), and the use of different funding allocations (committed vs disbursed, respectively).

The increase in funding between 2015 and 2018 (Fig 2) correlates to the Paris Climate Agreement in which health is enshrined as a right, and the establishment of the Green Climate Fund [44], which has contributed 22% of total health adaptation financing (2015–2019). EU Institutions were the top health donor and made 90% of their contributions in 2016 and 2017. The reason for the decrease in 2018 is not clear but could be tied to a lull in Green Climate Fund granting issuing. The Green Climate Fund’s initial resource mobilisation (IRM) was set to last from 2015 to 2018 [45]. Replenishment GCF-1 was completed by 2019, with programming allocation to be approved and funding disbursed between 2020 to 2023 [46].

With regards to our geographic analysis, the majority (61%) of the health adaptation projects were in Sub-Saharan Africa (SSA). This is promising as SSA has a high level of vulnerability to climate change and within the health sector. The World Bank estimates that by 2050 SSA will incur 80% of the global health costs from increased case burden of malaria and diarrheal disease [21]. For multilateral donors, 44% of projects and 48% of funding was targeting SSA. However, only two of the 10 ‘health principal’ projects were located in SSA. This indicates that although it appears that there are many health projects in SSA, these are mostly projects in which health was a co-benefit. 18 of the 20 countries with the highest health vulnerability score according to the ND-GAIN Index are in SSA. This suggests that a greater targeting of international climate finance for health should be directed towards these countries that experience the greatest need and vulnerability, including fragile and conflict affected states.

Based on the content analysis, we found that health is largely a co-benefit in projects. For example, many projects that focused on food or water security did not include a measured health indicator but mentioned expected health benefits such as increased health resilience, improved nutrition or reduced disease incidence as a potential consequential outcome of project activities. This aligns with the Lancet Countdown findings that the majority of approved ‘health related’ funding is directed at projects with potential secondary benefits for health [3,21,28–31,36]. Out of the ‘health significant’ projects, only 3 had concrete quantitative health indicators as part of their evaluation. The Green Climate Fund notes that the majority of their projects related to health comprise of mitigation projects with health co-benefits, and that studies evaluating the health co-benefits of climate policy and mitigation more broadly are becoming more prevalent [47]. However, including health indicators in the evaluation of individual projects is vital to the prioritisation of health in climate adaptation, especially if these projects are labelled as ‘health’ in databases. Secondary benefits to health are overall beneficial. However, if these outcomes are only assumed and not measured and monitored, the true benefit will remain unknown.

Additionally, if these projects are classified as relevant to health, this will lead to an overestimation of actual health-relevant projects. We found only 10 multilateral funded projects (in 2009–2019) that focused principally on health sector adaptation, worth USD 45.9 million (0.5% of total multilateral adaptation financing or 0.2% of total adaptation financing). This estimate is slightly higher than the Lancet Countdown’s estimation of USD 14.0 million (0.3% of total multilateral adaptation financing) and equivalent to a previous WHO estimate of USD 9 million (0.5% of total multilateral financing) [3,27]. This is also likely attributable to differences in methodology (timeframe, committed vs disbursed, definitions of health specific/health principal/health systems). Overall, across the different analyses, is it clear the volumes of adaptation finance related to health are low.

It is worth noting that analyses relying on donor self-reporting and tagging, or screening of project titles and abstracts only, may lead to an overestimation of the number of projects and volumes of financing targeting health [48,49] Our content analysis found only 10 projects out of the 50 projects tagged as “health” were directly tied to the health sector (i.e. health system capacity building) or the prevalence of health conditions (i.e. reducing vector-borne disease). Furthermore, for projects in which health is a co-benefit, the volume of adaptation finance does not solely target health. Project documents rarely quantified the breakdown of finance specifically directed towards health-related activities, rendering this more in-depth analysis impossible.

Significant gaps in health programming are apparent from this research. The majority of health adaptation projects in which there was a ‘principal’ health focus, were aimed at food security (with nutrition), followed by: health systems; surveillance and outbreak management; and clean drinking water supplies. Adaptation literature indicates that there is a need for more projects focussing on heat health action plans [8], mental health and psychosocial health (MHPH) [50], early warnings and early action plans [51], building climate resilient health infrastructure and systems [52], reducing global health inequity and the development of Health Action Plans as well as full implementation of health-climate strategies and plans [3,9,10,53]. The paucity of health indicators as part of project M&E criteria and the lack of emphasis on local adaptation were particularly significant findings. Moreover, there is growing recognition that community level health adaptation is missing from the global health adaptation financing agenda [54–56]. Building climate resilient and low carbon health systems was a key theme of COP26 in 2021 [38], and the breakthrough agreement on developing a new fund for loss and damage at COP27 in 2022 will have significant financial implications for highly climate vulnerable countries experiencing huge climate-linked losses and costs within the health sector [57]. The funding landscape will likely shift considerably from this baseline in the coming decade.

Historical demand for international climate finance from the health sector may be low for several reasons. Domestic government spending remains the largest source of funds for health [58], whilst international donor financing represents the smallest share of total financing for global health. However, an increasingly high proportion of development assistance for health (DAH) comes from non-governmental organisations, philanthropic organisations, public-private partnerships (such as Gavi and the Global Fund) and private corporations [59]. For example, in 2018 after the USA and the UK, the Bill & Melinda Gates Foundation was the third largest single contributor in terms of volume to development assistance for health [58]. Private sector investment, through both traditional and more innovative approaches such as blended financing (the use of development finance to mobilise additional private, commercial, or philanthropic finance), plays an important role in global health financing [23,60,61]. However, there is a significant data gap in the measuring and reporting of these private financial flows.

Low-income countries, which tend to have the highest health and climate vulnerability, rely the most on DAH whilst lower-middle income countries rely most on out-of-pocket spending and high-income countries on government and prepaid private spending [58]. Despite estimates that future health spending is projected to increase into the future, although at a slower rate of growth, global disparities in spending per capita will continue to persist [58]. Thus, while absolute levels of health spending are rising, they remain too low in many countries to finance essential health goals such as universal health coverage and climate adaptation [20].

Other reasons for low demand include the fact that climate finance has historically focused on sectors at high risk such as energy or agriculture, health ministries have historically lacked information on climate financing opportunities, and health investments do not lead to immediately obvious economic growth [60]. A 2019 survey conducted by the WHO found that a lack of information on opportunities, a lack of connection by health actors to climate change processes, and a lack of capacity to prepare country proposals were the top three challenges countries faced in accessing international climate finance for health [62]. These barriers may explain low levels of historic climate financing for health adaptation.

## Limitations

Despite providing a timely and useful expansion to previous work tracking climate adaptation finance flows to health activities, some limitations must be considered when interpreting the results. First, we were limited by the data available in both CFU and OECD-DAC databases. The OECD did not provide publicly available figures of disbursed funding, therefore, to be comparable between the databases we used committed funding amounts. This means our figures are overestimates of actual money spent in-country. It is possible that looking at disbursed flows would change the distribution of funds over time. OECD did not provide project duration and therefore we did not include project duration in the analysis. Additionally, the definitions of adaptation can vary, and the projects included in the study because of their categorisation as ‘adaptation’ relied on the OECD-DAC and CFU databases internal definition of adaptation. These may not have been commensurate with one another. The keywords search had several limitations. We picked keywords that were relevant to climate adaptation, including health impacts of climate change, health systems, and climate-sensitive infectious disease. We additionally searched keywords to health-relevant terms (e.g., agriculture, livelihoods, WASH) (see Table A in S1 Text), however, our scope and focus was on financing specific to the health sector and not to health-relevant sectors (such as agriculture). Our keywords search was restricted to English terms, therefore excluding non-English projects. CFU only had two projects that were not in English and neither project was health related. OECD had significantly more project entries not in English, particularly projects funded by French and German speaking countries. These were included if they were tagged using health sector codes. Only two projects were excluded from the content analysis for having non-English documentation. Most multilateral donors require English documentation. Although some projects would not have appeared in the keyword search, they would have been included via the sector codes and therefore included in the quantitative analysis. Additionally, some projects were missing information (such as the project title) or used broad categorizations (such as ‘global’ or ‘developing countries, unspecified’). Within the databases, the equivalent of USD 55 million was tagged as ‘global’ or ‘developing countries, unspecified’ and therefore were unaccounted for in regional calculations. Some projects are funded through partnerships between banks and receiving countries. However, only one donor is listed in the database. The amount of funding contributed by each donor could be inaccurate due to how it was entered into the database.

Furthermore, as the OECD and CFU databases track annual funding flows (meaning multi-year projects are entered multiple times), we had to make several assumptions during our data cleaning in order to analyse by project. We consolidated 153 entries under the assumption of tranche funding (see Methods), but we could not guarantee tranche funding for all of the projects reviewed when project documentation was not available. For example, we could not guarantee that USAID funded Peace Corp projects (n = 67) were tranche funding. Therefore, our findings show that the United States funded significantly more projects (n = 128) than any other donor but had much lower average funding per project. Lastly, it was not possible to locate the majority of project documents for bilateral projects, so our content analysis was limited to available multilateral project documentation. Often, some form of documentation was available (project website, annual report) however these documents lacked necessary information including funding amounts, co-financing, project duration, and project evaluation indicators. This was true even for bilateral donors that offer project documentation on their website (i.e., of the Norwegian Agency for Development Cooperation’s (NORAD) 23 projects, only 8 appeared in their project search feature and none had programme documents). Bilateral projects were excluded from the content analysis due to difficulties in retrieving official documentation. Therefore, it is likely that there is an overestimation on the volumes of bilateral funding towards health.

## Conclusion

The results of this study provide baseline estimates of volumes of financing and geographic targeting that can help governments, donors, and researchers identify existing shortfalls and gaps. Moving forward, future research—and, importantly, action—must focus on the solutions to reduce these gaps. Overall, volumes of international climate adaptation finance targeting the health sector are low, but international climate finance is only one source of funding for the health sector. How best to finance climate resilient and sustainable health systems will be a key area of future health economic and policy research, and links strongly with the universal health coverage (UHC) agenda. In addition, we found that the majority of projects with health as their principal focus tended to target national systems with little emphasis on local adaptation. A deeper understanding of the most effective ways of empowering local health adaptation is needed. Additionally, if health becomes a cross-cutting theme across climate adaptation programming, incorporating health indicators in projects across sectors will be vital. Identifying the most appropriate indicators as part of the monitoring and evaluation process will be a significant area of future research and can contribute to quantifying assumed health benefits. Lastly, in order to support accurate recording and research on climate finance for health, we recommend more stringent criteria for sector tags when countries are reporting projects. Ensuring accurate labelling of health projects will allow for easier checks of financial flows and can reduce the risk of overestimation. Furthermore, increased transparency from bilateral funders, including easy access to project proposal and evaluation documents will also facilitate third parties’ ability to verify and cross-check funding flows and support the expansion of climate finance research.

The mobilisation of climate adaptation finance is an important mechanism by which to address climate change impacts on health in the most vulnerable countries. As part of this, it is essential to systematically track funding flows and progress in order to establish a baseline and better direct the use of scarce public resources. This study systematically searched international financial reporting databases (OECD and CFU) to identify climate change adaptation projects related to the health sector between 2009 and 2019. Our findings show that volumes of adaptation finance related to health are low: in a decade of funding (2009–2019), USD 1,431 million (4.9%) of multilateral and bilateral international adaptation finance has been committed to health activities. Our content analysis of projects tagged as ‘health’ found that only 10 projects out of 50 potential projects were directly tied to the health sector via the main project aim, objectives, and metrics. This suggests that multilateral donors (such as GCF, GEF, Adaptation Fund) only provide <0.5% of total multilateral adaptation financing or 0.2% of total adaptation financing to health sector adaptation. Even this may be an overestimation, as publicly available project documents rarely quantified the breakdown of finance directed to different health activities. Adaptation in the health sector alone will have limited impact as health is reliant on complex interaction of environmental and social determinants. Nonetheless, it is a critical sector for adaptation as climate change’s impact on health and health systems has compounding impacts across manifold sectors. These effects are already being felt and are disproportionately affecting millions of those who have contributed the least to the problem.

## Supporting information

S1 Text Table A. Number of adaptation projects retrieved by health sector specific key word. Table B. Number of adaptation projects retrieved by health-relevant keywords (projects were not included in subsequent analysis). Table C. Number of health adaptation projects by donor. Table D. Health adaptation projects by channel of delivery type (i.e. implementing agency). Table E. Categorization of multilateral projects included in the qualitative content analysis. Projects could be entered more than once in this matrix if there were multiple project components.
(DOCX)

S1 Data Health adaptation projects (2009–2019) dataset. (XLSX)
