# Supplementary material for: How much bilateral and multilateral climate adaptation finance is targeting the health sector? A scoping review of official development assistance data between 2009–2019
Source: PLOS Glob Public Health. 2023 Jun 14;3(6):e0001493. doi: 10.1371/journal.pgph.0001493 (PMC10266659; doi:10.1371/journal.pgph.0001493)
Supplement: S1 Text — Table A. Number of adaptation projects retrieved by health sector specific key word. Table B. Number of adaptation projects retrieved by health-relevant keywords (projects were not included in subsequent analysis). Table C. Number of health adaptation projects by donor. Table D. Health adaptation projects by channel of delivery type (i.e. implementing agency). Table E. Categorization of multilateral projects included in the qualitative content analysis. Projects could be entered more than once in this matrix if there were multiple project components. (DOCX) [file pgph.0001493.s001.docx]

Supplementary Material

Table of Contents

[‘Adaptation’ definition 1](#_Toc122356449)

[Key word search results 1](#_Toc122356450)

[Breakdown of projects by donor and channel of delivery 2](#_Toc122356451)

[Multilateral Content Analysis Project Topic Area 5](#_Toc122356452)

# ‘Adaptation’ definition

According to OECD-DAC Database: ‘Adaptation’ tagged projects must “intend to reduce the vulnerability of human or natural systems to the current and expected impacts of climate change, including climate variability, by maintaining or increasing resilience, through increased ability to adapt to, or absorb, climate change stresses, shocks, and variability and/or by helping reduce exposure to them. This encompasses a range of activities from information and knowledge generation, to capacity development, planning and the implementation of climate change adaptation actions.”[42]

# Key word search results

In addition to the retrieval of adaptation projects via the sector tag provided by the databases, we ran a secondary back-up key word search to ensure that all health projects had been included. The tables below summarise the number of projects in which the keyword was present in the title or abstract. Table A provides information on the keywords which were selected as directly of relevance to the health sector (resulting in the 509 projects which were included in the study analysis). Table B provides information on the keywords used which were of significant relevance to the health sector (e.g. sanitation, water, agriculture). Given the high number of projects returned for this search we chose to not include this data in our analysis as our objective was to estimate climate adaptation financing which was directed at the health sector including via a qualitative review of publicly available documentation. We would not have been able to review such a high volume of project documentation by including these health-relevant projects in the content analysis. However, once we began the document retrieval for the content analysis we were limited by the lack of publicly available documentation for bilaterally funded projects. Interested researchers are encouraged to contact the author team should they wish to access the dataset with both the health specific key words and the health-relevant key words.

**Table A. Number of adaptation projects retrieved by health sector specific key word.**

| Key word | Number of projects n (%) |
| --- | --- |
| Health | 349 (68.57) |
| Epidemic | 1 (0.20) |
| Surveillance | 3 (0.59) |
| Disease | 14 (2.75) |
| Mortality | 3 (0.59) |
| Hospital | 4 (0.79) |
| Nutrition | 104 (20.43) |
| Malnutrition | 2 (0.39) |
| Psychosocial | 1 (0.20) |
| Waterborne (diarrheal) | 1 (0.20) |
| Vectorborne (malaria) | 2 (0.39) |
| Vectorborne (dengue) | 1 (0.20) |
| Medical | 3 (0.59) |
| Air Quality (pollution) | 10 (1.96) |
| Heat | 11 (2.16) |
| Total | 509 (100) |

**Table B. Number of adaptation projects retrieved by health-relevant keywords** **(projects were not included in subsequent analysis).**

| Keyword | Number of projects n (%) |
| --- | --- |
| Sanitation | 1221 (24.81) |
| Water | 213 (4.33) |
| Ecosystem | 127 (2.58) |
| Migration | 50 (1.02) |
| Agriculture | 2328 (47.30) |
| Food Security | 167 (3.39) |
| Total | 4922 (100) |

# Breakdown of projects by donor and channel of delivery

Table C provides the full breakdown of health adaptation projects by donor which appears in the manuscript for only the top 20 donors, ranked.

**Table C. Number of health adaptation projects by Donor.**

| Donor | Number of health projects  n (%) |
| --- | --- |
| Australia | 17 (3.34) |
| Austria | 5 (0.98) |
| Canada | 46 (9.04) |
| Czech Republic | 3 (0.59) |
| EU Institutions (excl. EIB) | 12 (2.36) |
| Finland | 7 (1.38) |
| France | 12 (2.36) |
| Germany | 35 (6.88) |
| Hungary | 1 (0.20) |
| Iceland | 2 (0.39) |
| Ireland | 81 (15.91) |
| Italy | 4 (0.79) |
| Japan | 9 (1.77) |
| Korea | 4 (0.79) |
| Netherlands | 1 (0.20) |
| Norway | 23 (4.52) |
| Portugal | 1 (0.20) |
| Spain | 22 (4.32) |
| Sweden | 12 (2.36) |
| Switzerland | 10 (1.96) |
| United Arab Emirates | 1 (1.96) |
| United Kingdom | 21 (4.13) |
| United States | 128 (25.15) |
| Adaptation for Smallholder Agriculture Programme (ASAP) | 3 (0.59) |
| Adaptation Fund (AF) | 15 (2.95) |
| Climate Investment Fund (CIF) | 1 (0.20) |
| UN Food and Agriculture Org (FAO) | 6 (1.18) |
| Global Environment Facility (GEF) | 1 (0.20) |
| Green Climate Fund (GCF) | 12 (2.36) |
| International Fund for Ag Development (IFAD) | 4 (0.79) |
| Least Developed Countries Fund (LDCF) | 4 (0.79) |
| Nordic Development Fund (NDF) | 1 (0.20) |
| Special Climate Change Fund (SCCF) | 5 (0.98) |
| Total | 509 (100) |

**Table D. Health adaptation projects by channel of delivery type (i.e. implementing agency).**

| Implementing Agency | Number of health adaptation projects  n (%) |
| --- | --- |
| Central government | 22 (4.32) |
| Development bank | 16 (3.14) |
| Developing country-based NGO | 143 (28.09) |
| Donor Government | 62 (12.18) |
| Government | 3 (0.59) |
| Government department | 2 (0.39) |
| Intergovernmental organization | 75 (14.73) |
| International NGO/NGO | 23 (4.52) |
| Local Government | 2 (0.39) |
| Not specified | 4 (0.79) |
| Other | 47 (9.23) |
| Private sector in provider country | 11 (2.16) |
| Private sector in third country; central government | 1 (0.20) |
| Private Sector Institutions | 2 (0.39) |
| Public Sector Institutions | 4 (0.79) |
| Recipient Government | 30 (5.89) |
| Third Country Government (Delegated co-operation) | 6 (1.18) |
| University, college or other teaching institution, research institute or think-tank | 56 (11.00) |
| Total | 509 (100) |

# Multilateral Content Analysis Project Topic Area

Table E presents the major themes or topic areas (e.g. capacity building, early warning system) covered by the multilateral adaptation projects that had either been tagged as ‘health’ or contained a health sector specific keyword in their title or abstract. Projects were excluded for the qualitative analysis if they did not have a direct health-specific objective (i.e., the health objective was not explicitly indicated in the activity documentation), if project documentation could not be retrieved, or if the documentation was not in English (see Limitations). During the content analysis of the publicly available documentation we recorded the major themes or focus of the project and categorized whether the project has health as a principal focus or a significant focus. As per the methods, Following this data extraction, projects were then classified as health principal, health significant, or not health focused. Projects were tagged as health principal if the main project aim, objectives, and metrics were directly tied to the health sector (i.e., health system capacity building) or the prevalence of health conditions (i.e., reducing vector-borne disease). Projects were tagged as health significant if the main project aim, objectives, and metrics were not directly tied to the health sector, but a strong correlation was made between the activities and a health outcome. The health outcome should be both defined and measured in order for the project to be tagged as health significant. For example, livelihood and agricultural programming that showed a clear, measured relationship between the activities and a reduction in food insecurity or malnutrition would be tagged as health significant. Projects were tagged as not health focused if a connection was not made between the main program activities and a health outcome. For example, a livelihood programme that did not make the connection between the programme activities and a measured potential health outcome, irrespective of whether it could have a potential health benefit, was tagged as not health focused.

**Table E. Categorization of multilateral projects included in the qualitative content analysis. Projects could be entered more than once in this matrix if there were multiple project components.**

|  | Health Principal | Health Significant | Not Health Focused | Total |
| --- | --- | --- | --- | --- |
| Agriculture | 0 | 2 | 4 | 4 |
| Animal health | 0 | 0 | 2 | 2 |
| Capacity building | 8 | 0 | 0 | 8 |
| Early warning systems | 6 | 0 | 0 | 6 |
| Surveillance | 7 | 0 | 0 | 7 |
| Health national adaptation plans | 6 | 0 | 0 | 6 |
| Knowledge/training | 2 | 0 | 0 | 2 |
| Disaster risk reduction | 0 | 1 | 1 | 2 |
| Ecosystem health | 0 | 0 | 2 | 2 |
| Food security/nutrition | 0 | 2 | 0 | 2 |
| Infectious disease | 1 | 1 | 0 | 2 |
| Infrastructure | 0 | 1 | 0 | 1 |
| Land management | 0 | 0 | 2 | 2 |
| Livelihoods | 0 | 11 | 3 | 14 |
| National adaptation plan implementation | 0 | 0 | 1 | 1 |
| Ocean ecosystem | 0 | 0 | 1 | 1 |
| WASH | 0 | 5 | 1 | 6 |
| Water infrastructure | 0 | 3 | 0 | 3 |
